# Downregulation of miR-375 contributes to ERBB2-mediated VEGFA overexpression in esophageal cancer

**DOI:** 10.7150/jca.63836

**Published:** 2021-10-20

**Authors:** Shuchang Ren, Xiaohui Tan, Melinda Z. Fu, Shuyang Ren, Xiaoling Wu, Tao Chen, Patricia S. Latham, Paul Lin, Yan-gao Man, Sidney W. Fu

**Affiliations:** 1 Department of Medicine, Division of Genomic Medicine, and Department of Microbiology, Immunology and Tropical Medicine, The George Washington University School of Medicine and Health Sciences, Washington, DC.; 2 Department of Medicine, Chengdu Military General Hospital, Chengdu, Sichuan, China.; 3 Department of Pathology, The George Washington University School of Medicine and Health Sciences, Washington, DC.; 4 Department of Surgery, The George Washington University School of Medicine and Health Sciences, Washington, DC.; 5 Department of Pathology, Hackensack Meridian Health-Hackensack, University Medical Center, Hackensack, NJ; the International Union for Difficult to treat Diseases (IUDD), Silver Spring, MD.

## Abstract

Esophageal cancer (EC) is a lethal cancer with an extremely aggressive nature and poor survival rate. However, the molecular mechanisms driving the occurrence and progression of EC are not well understood. MicroRNAs (miRNAs) are small RNA molecules that regulate the expression of protein-coding genes. miRNA-mediated gene regulation plays an important role in EC. By cross-referencing studies from NCBI, we found that microRNA-375 (miR-375) is one of the most frequently downregulated miRNAs in EC. We assessed expression of miR-375 in EC cell lines and primary EC tissues and their matched normal tissues. We found significant downregulation of miR-375 in both cell lines and EC tissues. Forced expression of miR-375 attenuated EC cell proliferation and invasion. Human epidermal growth factor receptor 2 (HER2, ERBB2), a known proto-oncogene, was identified here as one of the potential target genes of miR-375. Ectopic expression of miR-375 significantly suppressed the expression of ERBB2 and subsequently downregulated one of its target genes, vascular endothelial growth factor A (VEGFA), which is related to cancer invasion and metastasis. These findings suggest that miR-375 acts as a tumor suppressor by blocking the ERBB2/VEGFA pathway with the potential to modulate the occurrence and/ or progression of EC.

## Introduction

Esophageal cancer (EC) is one of the deadliest cancers worldwide 1, 2. It consists of two common histologic types: esophageal squamous cell carcinoma (ESCC), which accounts for 80% of EC cases worldwide 3, and esophageal adenocarcinomas (EAC), which has a higher incidence in Western world 4. Due to fact that most patients have distant metastases from EC at the time of diagnosis, the prognosis remains poor. The average five-year overall survival rates of the two type tumors are approximately 15% 5. However, the molecular mechanism of EC is not well understood. MicroRNAs (miRNAs) are small RNA molecules that regulate the expression of protein-coding genes by directly binding to target mRNAs in a sequence-specific manner. miRNAs are present in tissue, blood and other body fluids and have emerged as critical components of complex functional pathways involved in carcinogenesis. Specific miRNAs have been identified to be aberrantly expressed in ECs and found to correlate with diagnosis, prognosis and response to chemotherapy 6. However, little is known about the cellular function of these differently expressed miRNAs.

By cross-referencing our RNASeq data (data not shown) with others' from NCBI, we found that miR-375 is one of the most frequently downregulated miRNAs in EC. The goal of this study was to investigate the mechanism of miR-375 dysregulation in EC.

## Materials and Methods

### Cell lines

ESCC cell lines, KYSE-70 and KYSE-180 were cultured in RPMI 1640 medium supplemented with 10% FBS and 1% penicillin and streptomycin antibiotics. EAC cell lines, FLO-1 and JHU-ad1, were cultured in Dulbecco's Modified Eagle Medium (DMEM) with 10% FBS and 1% penicillin and streptomycin antibiotics. These four cell lines were kindly provided by Dr. Stephen J. Meltzer (Johns Hopkins School of Medicine). The non-cancerous epithelial esophageal cell line HET-1A purchased from ATCC, was cultured in the base medium for this cell line (BEBM) along with BEGM kit (Catalog No. CC-3170, Lonza) with no GA-1000 (gentamycin-amphotericin B mix). All the cell lines were incubated in a 37˚C humidified incubator with 5% CO2.

### Clinical samples and tissue microdissection

The formalin-fixed, paraffin-embedded (FFPE) EC specimens were retrieved from the archive of the Chengdu Military General Hospital. None of the patients had received any chemotherapy or radiotherapy before sampling. The diagnosis of the archived esophageal cancer samples was confirmed by a second pathologist independently (Table 1). The FFPE tissues were microdissected into the following components, adjacent normal esophageal epithelium, dysplasia and carcinoma as described previously 7. All sample collections were under the ethical standards of the institutional and national research committee and in agreement with the Helsinki Declaration and its later amendments or comparable ethical standards.

### RNA extraction and quantitative real-time reverse transcription-PCR (qRT-PCR)

Total RNA from cultured cells and FFPE samples was isolated and quantitated as described previously 8. miR-375-3p (Acc#: MIMAT0000728) expression was assayed by qRT-PCR using the Taqman MiRNA Reverse Transcript Kit (Thermo Fisher Cat# 4366596) with primer (5'- UUUGUUCGUUCGGCUCGCGUGA-3' Cat# 4427975). The target gene ERBB2 (F: 5'-CAGTGCAGCACAGAGACTCA-3', R: 5'-CCGGTGCACACTCACTTTTG-3') and its downstream gene VEGFA (F: 5'-ACAAATGTGAATGCAGACCAAA-3', R: 5'-ACCAACGTACACGCTCCAG-3') were analyzed using RT Real-Time™ SYBR Green (Bio-Rad Laboratories) as described previously 9.

### Protein extraction and Western blotting

Protein was extracted using the RIPA lysis buffer (ThermoFisher) according to the manufacturer's protocol. Briefly, proteins isolated from cells were separated electrophoretically. Equal amounts proteins (50 µg) were run on 12 % SDS-polyacrylamide gels and transferred onto nitrocellulose membrane. The following antibodies were used: anti-rabbit HER-2 (PA5-14635, ThermoFisher), anti-rabbit VEGF (PA5-16754, ThermoFisher), anti-mouse Phospho-AKT1 (44621G, ThermoFisher), anti-rabbit beta actin (PA1-183, ThermoFisher). Western blot analysis with chemiluminescent detection was performed as described 10.

### MiRNA and plasmid Transfection

EC cells were seeded (10^6^ cells/well) in a 12-well plate in antibiotic-free medium for 24 h prior to transfection to achieve 80% confluency at the time of transfection. Transfection was conducted by the Lipofectamine RNAiMAX (Life Technologies, Cat# 13778) delivery of miRNA precursors, including miR-375 mimic, inhibitors and their mock controls (ThermoFisher) using the Opti-MEM Reduced Serum Medium (Life Technologies). For rescue experiments, the pHAGE-ERBB2 plasmid containing full-length human ERBB2 cDNA without 3′UTR was purchased (Addgene, Cat# 116734). The EC cell lines transfected with miR-375 mimic and inhibitor were then co-transfected with pHAGE/ERBB2 plasmid or empty pHAGE vector using the FuGENE reagent (Promega). The transfected cells were subjected to further analysis after 48h post transfection.

### MTT assays

For MTT assay, the cells transfected with miR-375 mimic, inhibitor or their corresponding mock controls were washed with 1xPBS. MTT working solution (5 mg/ml stock MTT diluted in optiMEM to 0.5 mg/ml working solution) was added to each well and incubated at 37°C for 3 h. MTT solution was then removed before adding 100 μl of DMSO to each well for additional 30 min incubation. Color development was measured using a spectrophotometer at 490 nm on a plate reader (BIO-TEK Instruments) and quantified as per the manufacturer protocol (Promega, USA).

### Matrigel invasion assays

EC Cell invasion capability was evaluated by Matrigel invasion assays using the BD BioCoat™ Matrigel™ Invasion Chamber (BD Biosciences) according to the manufacturer's instructions as previously described 11. Briefly, 500 μl of warm (37°C) serum-free DMEM medium was added to the upper and lower chambers and allowed to rehydrate for 2 h in a 37 °C cell culture incubator, while 8 x 10^4^ cells transfected by either miR-375 mimic or inhibitor with the mock controls for 24 h, were seeded onto the top chamber of pre-wetted inserts. Cells were incubated in the Matrigel chamber in a 37 °C humidified incubator with 5% CO_2_ for 48 h. The non-invading cells were removed by scrubbing from the upper surface of the membrane with a cotton swab. The invasive cells present were fixed, stained with the Diff-Quick staining solution and counted (five microscope fields under the 10X len). Experiments were done in duplicates for each cell line twice. Cell counts were performed on five non-overlapping random fields for each chamber and four chambers were counted for each experimental using.

### Dual luciferase reporter assay

For the luciferase reporter assay, 2 × 10^5^ cells/well were plated in a 24-well plate. After 24 h incubation in a 37 °C cell culture incubator, cells were co-transfected with 100 ng of pEZX-ERBB2-3′UTR (wild type and mutant) expression clones inserted downstream of a secreted Gaussia luciferase (GLuc) reporter and 100 ng of DNA with pEZX-miR-375 or the pEZX-MT scrambled control using the FuGENE Transfection Reagent (Promega). Luciferase activities were determined with the Secrete-PairTM Dual Luminescence Assay Kit (Genecopoeia)*.* GLuc luciferase activities were normalized to SEAP luciferase expression for each sample.

### Statistical analysis

The difference of miR-375 expression in clinical samples was analyzed by the exact two-sided binomial test. Data were expressed as mean ± standard error (S.E.). MTT assays between control and miR-375 mimic or inhibitor transfected groups were analyzed by Permutation tests. Matrigel assay between control and miR-375 or inhibitor transfected groups was analyzed using the student's t-test (two tailed). *P* value less than 0.05 was considered statistical significance.

## Results

### Decreased expression of miR-375 in EC cell lines and clinical samples

**Figure 1:**
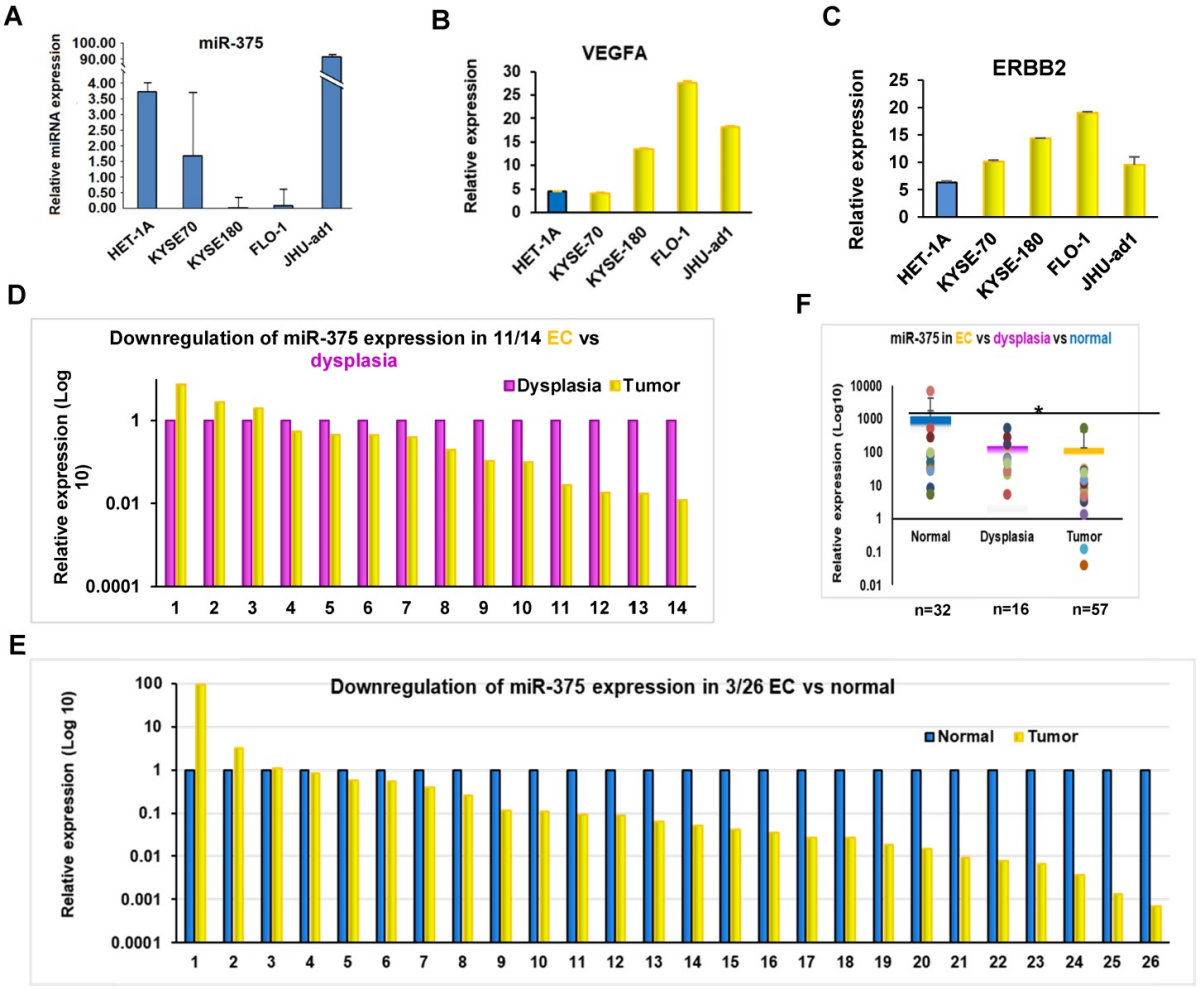
**Expression of miR-375 and its target genes in EC**. **A**. Expression of miR-375, ERBB2 and VEGFA in EC cell lines by qRT-PCR. Expression of miR-375 in EC cell lines are significantly decreased compared to HET-1A, a non-cancerous epithelial esophageal cell line. **B and C.** An inverse correlation between miR-375 and ERBB2/VEFGA expression pattern were observed in EC cell lines. **D.** Decreased miR-375 in pure population of tumor and dysplasia cells microdissected from FFPE tissue. The level of miR-375 expression was significantly decreased in 11 of 14 ESCC compared to their adjacent dysplasia. **E.** The level of miR-375 expression was significantly reduced in 23 of the 26 in ESCC compared with the adjacent normal tissues. F. Expression of miR-375 in normal, dysplasia and EC cells. Values represent the mean ± S.D. from three independent experiments. (*p <0.05).

To determine the expression of miR-375 in EC, we first measured the expression of miR-375 in EC cell lines by qRT-PCR. The expression levels of miR-375 in the four EC cell lines were lower than that in the non-cancerous epithelial esophageal cell line HET-1A (Fig. 1A). We then examined the expression of miR-375 in archival FFPE specimens, which were microdissected in pure populations of normal, dysplasia and tumor cells. Consistent with the findings in the cell lines, miR-375 was significantly decreased in 11 of 14 (78.6%) ESCC compared to their adjacent areas of dysplasia and in 23 of 26 (88.5 %) ESCC compared to their adjacent normal tissues, respectively (Fig. 1D-F). These results suggest a high prevalence of downregulation of miR-375 during the progression of EC.

### miR-375 inhibits cell proliferation in EC

**Figure 2:**
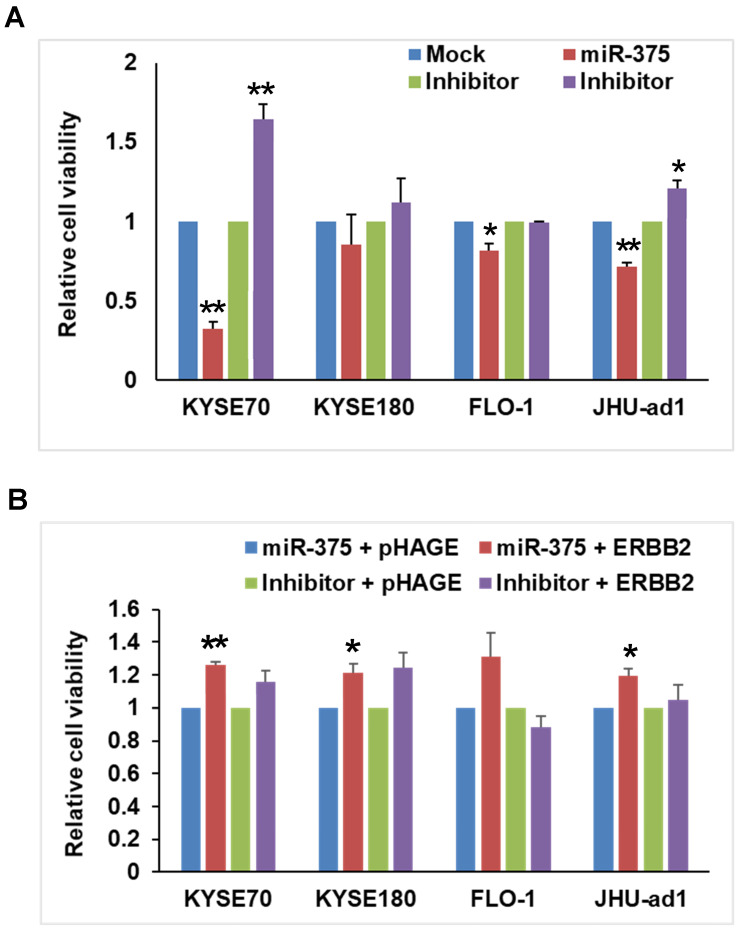
** miR-375 inhibits cell proliferation that rescued by re-expression of ERBB2**. **A.** miR-375 inhibits cell proliferation. The EC cell lines were transfected with mock control, miR-375 mimic, inhibitor control and miR-375 inhibitor; and cell viability was measured by MTT assay. Transfection of miR-375 mimic significantly reduced the proliferation compared to that of mock control in EC cell lines. Values represent the mean ± S.D. from three independent experiments. (** p < 0.01, *p <0.05). **B**. Rescue experiment showed that the anti-proliferative effect of miR-375 is reversed by ERBB2 restoration. The pHAGE-ERBB2 cDNA expression vectors were transfected into EC cells after 48h of miR-375 mimic or inhibitor transfection. The effect of ERBB2 restoration on cell proliferation in miR-375 transfected cell lines was measured by MTT. The cell proliferation was significantly increased after transfection of ERBB2 into miR-375 transfected or cells compared to that of pHAGE empty control in KYSE-70, FLO-1 and JHU-ad1 cells and KYSE-180, although the change was not statistically. Values represent the mean ± S.D. for three independent experiments. (** p < 0.01, *p <0.05).

After confirming the downregulation of miR-375 in EC, we sought to determine the functional role of miR-375 in EC. We firstly transfected miR-375 to EC cell lines and examined proliferation using MTT assays. Ectopic expression of miR-375 significantly inhibited the proliferation compared to the mock control in ESCC cell lines KYSE-70, EAC cell lines FLO-1 and JHU-ad1. Overexpression of miR-375 also inhibited the proliferation in ESCC cell line KYSE-180, although the change was not statistically significant. Conversely, transfection of miR-375 inhibitor significantly increased cell proliferation in ESCC cell line KYSE-70 and EAC cell line JHU-ad1 compared to transfected mock inhibitor. Transfection of miR-375 inhibitor also increased cell proliferation in ESCC cell line KYSE-180, although the change was not statistically significant (Fig. 2A). These results indicate a proliferative effect of miR-375 in EC cell lines.

### miR-375 inhibits cell invasion in EC

**Figure 3:**
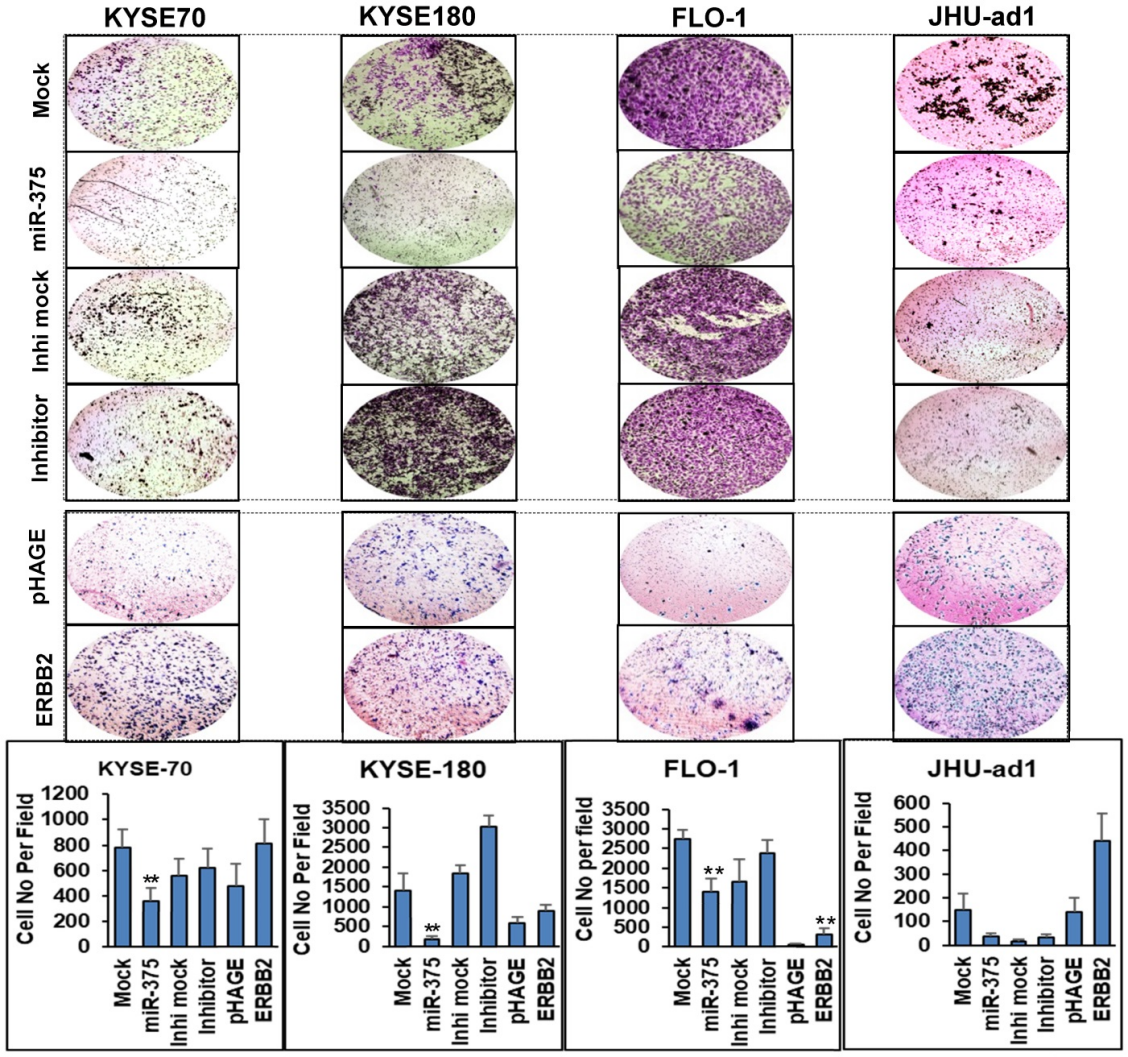
** miR-375 inhibits invasive ability of EC cell lines that rescued by re-expression of ERBB2.** Transwell assays were performed for the invasive activity of EC cells transfected with either miR-375 mimic or the inhibitor with their mock controls. Overexpression of miR-375 significantly reduced cell invasion in ESCC cell lines KYSE-70 and KYSE-180 and in EAC cell line FLO-1 but not significant in JHU-ad1 cells. Re-expression of ERBB2 resulted in significantly increased invasive ability compared to the pHAGE empty vector transfected one in EC cell lines. Invasive ability of the cells was displayed as a percentage of the absolute cell numbers (bottom). Results are displayed as mean data ± SE. (** p < 0.01, *p <0.05). Five fields of unit area on each membrane or whole membrane were counted for cell numbers, and the experiments were repeated three times with triplicates.

To test the impact of miR-375 on invasion, we performed Matrigel invasion assays in EC cell lines. Consistent with the effect of miR-375 on EC cell proliferation, a significantly decreased invasive capability was observed in miR-375-transfected ESCC cell lines KYSE-70, KYSE-180 and EAC cell line FLO-1, also in EAC cell line JHU-ad1 although the change was not statistically significant compared to the mock transfections. Conversely, transfection of miR-375 inhibitor significantly increased invasion capability compared to the mock inhibitor transfection in ESCC cell lines KYSE-180 and slightly increased invasion capability in KYSE-70 , FLO-1 and JHU-ad1, although the change was not statistically significant (Fig. 3).

### miR-375 directly targets ERBB2 in EC

**Figure 4:**
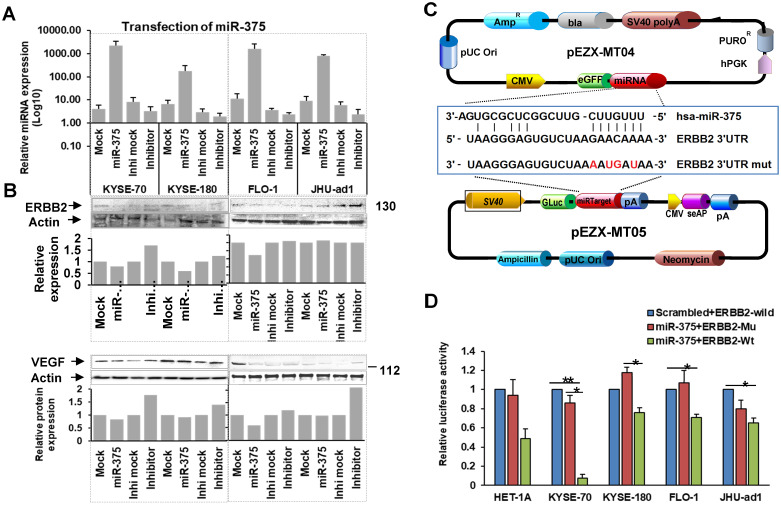
** miR-375 regulates ERBB2 and VEGFA in EC**. **A.** Expression of miR-375 after transfection. **B.** Forced expression of miR-375 in EC cell lines resulted in decreased ERBB2 and VEGFA expression. **C.** Map of the plasmids pEZX-MT04 containing miR-375, and pEZX-MT05 containing 3'-UTR of ERBB2 to illustrate the binding site of miR-375 at the 3'-UTR of ERBB2, and its mutant control sequence. **D.** Dual luciferase reporter assay. Co-transfection with pEZX-miR-375 and pEZX-ERBB2 3' UTR wild type significantly decreased the luciferase activities compared to that with pEZX-miR-375 and ERBB2 3' UTR mutant/miR-375 scrambled control and ERBB2 3' UTR wild type sequence in ESCC cell lines. The data were reported as mean ± S.D. from three independent experiments (** p < 0.01, *p <0.05)

Using TargetScan and microrna.org bioinformatics platforms, we identified a list of potential target genes of miR-375, including ERBB2. Since ERBB2 gene plays an important role in human malignancies and a high prevalence of ERBB2 expression has been demonstrated in EC 12, we focused on the regulatory role of miR-375 over ERBB2. We observed an inverse correlation between miR-375 and ERBB2 expression in EC cell lines (Fig. 1A and B). Furthermore, miR-375 transfection (Fig. 4A) resulted in significant ERBB2 downregulation compared to the mock transfection, while miR-375 inhibitor transfection restored the expression of ERBB2 compared to the inhibitor mock transfection in ESCC cell lines, KYSE-70, KYSE-180, and EAC cell line FLO-1, but not in JHU-ad1 (Fig. 4B). Our results suggest that miR-375 suppresses ERBB2 expression.

To confirm ERBB2 is a direct target of miR-375, we performed luciferase reporter assays by co-transfecting pEZX-MT05 vector containing HER2 3′UTR region with the miR-375 binding site (either wild type or mutant sequences), and pEZX-MT04 vector containing miR-375 (Fig. 4C) or scrambled control. The luciferase activities were significantly decreased in ESCC cell lines, KYSE-70 and KYSE-180 co-transfected with the pEZX-MT04 vectors containing miR-375 plus pEZX-MT05 vector containing ERBB2 3′UTR wild type sequence, compared to that co-transfection of either miR-375 plus 3′UTR mutant sequence or scrambled control plus ERBB2 3′UTR wild type one. Decreased luciferase activity was also observed in EAC cell lines, FLO-1 and JHU-ad1 co-transfected with miR-375 plus ERBB2 3′UTR wild type compared to that co-transfected with miR-375 plus scrambled control (Fig. 4D). These results suggest that miR-375 directly regulates ERBB2 by binding to its 3′UTR in EC especially in ESCC.

### miR-375 suppresses ERBB2 mediated VEGF expression in ESCC

The role of ERBB2 in angiogenesis has been well established and ERBB2 overexpression in human tumors is closely associated with increased VEGF 13, 14, 15, 16, which is well known to be related to invasion and metastasis of malignant tumors including EC 17, 18, 19. Furthermore, ERBB2 has been implicated in the regulation of VEGF 20, 21. We observed an inverse correlation between miR-375 and ERBB2 expression as well as miR-375 and VEGFA expression in EC cell lines (Fig. 1A, B and C). As there are no predicted miR-375 binding sites at VEGFA mRNA 3'UTR, we hypothesize that miR-375 indirectly inhibits the expression of VEGF by suppressing ERBB2. In fact, ectopic transfection of miR-375 repressed not only ERBB2 but also VEGFA expression with significantly in ESCC but not in EAC cell lines. This result suggests that miR-375 suppresses ERBB2-mediated VEGF expression in ESCC.

### Tumor suppressive effects of miR-375 on ESCC cell lines were reversed by restoration of ERBB2 expression

To verify whether miR-375 suppresses ESCC tumor growth by targeting ERBB2, a rescue experiment was conducted by transfecting pHAGE-ERBB2 plasmid containing full-length human ERBB2 cDNA into miR-375 transfected EC cell lines. As expected, the cell proliferation was significantly increased after transfecting pHAGE-ERBB2 into miR-375-transfected cell lines, KYSE-70, FLO-1 and JHU-ad1, compared to that of pHAGE empty vector control. For KYSE-180 cells, proliferation of the cells was also increased but not statistically significant (Fig. 2B). ERBB2 expression was reactivated in miR-375 inhibitor transfected EC cell lines, KYSE-70, KYSE-180 and JHU-ad1, although the change was not statistically significant (Fig. 2B).

To determine the effect of miR-375-mediated ERBB2 expression on cell invasion, we performed Matrigel invasion assay. Re-expression of ERBB2 enhanced the invasion capability compared to the pHAGE empty vector transfected one in EC cell lines (Fig. 3). In addition, Western blot results showed that re-expression of ERBB2 partially increased VEGF expression. These results suggest that miR-375 act as a tumor suppressor by inhibiting ERBB2 and/or ERBB2-mediated VEGF expression.

## Discussion

To date, dysregulation of miR-375 has been reported in a variety of cancers with different functions. Downregulated miR-375 was reported in the majority of cancers, such as oral squamous cell carcinoma 22, laryngeal squamous cell carcinoma 23, head and neck cancer 24, breast cancer 25, lung cancer 26, liver cancer 27, gastric carcinoma 28, colorectal cancer 29 and pancreatic cancer 30. Upregulated miR-375 was mainly reported in urogenital carcinomas such as prostates cancer 31, ovarian cancer 32 and cervical cancer 33. However, there are no reports about the regulation of miR-375 on ERBB2/VEGF pathway in EC to the best of our knowledge. We found decreased miR-375 expression in EC tissue as well as pre-cancerous dysplasia. miR-375 is located on chromosome 2q35 34, which is often deleted in EC 35, suggesting that the downregulation of miR-375 may be due to genomic DNA deletion in human EC. If so, it is possible that decreased levels of miR-375 may serve as a potential diagnostic marker for the early detection of EC.

ERBB2 proto-oncogene is a type I transmembrane tyrosine kinase growth factor receptor that plays a key role in the processes of tumor cell proliferation, differentiation and growth 36. Overexpression of HER2 in human tumor cells is closely associated with increased VEGF expression 13.

Overexpression and/or amplification of ERBB2 and VEGF have been found in a variety of human cancers, including EC 12, 18, 19, 37-45. ERBB2 and VEGF are located on chromosome 17q12 and 6p21.1, respectively. As both regions are frequently amplified in EC 46, 47, ERBB2 and VEGF overexpression can be therapeutic targets for GI cancer. In fact, combined blockade of both HER2 and VEGF exerts synergistic tumor growth inhibition in gastroesophageal cancer 48. In this study, we found that decreased miR-375 is often associated increased ERBB2/VEGF expression in EC. Our data demonstrate that ERBB2 is a direct target of miR-375 and VEGFA can be indirectly targeted by miR-375, implying that frequent overexpression of ERBB2 and VEGF may be, at least in part, due to downregulation of miR-375 in the development of EC. We further demonstrate that, overexpression of miR-375 significantly suppressed proliferation and invasion in EC cells. These findings suggest that miR-375 is potentially an important tumor suppressor in the initiation and/or progression of EC by targeting ERBB2/VEGF. The miR-375/ ERBB2/VEGF axis might serve as a signature for both diagnostic and therapeutic target in EC. Both drugs that specifically target ERBB2 and VEGF have been shown to confer clinical benefit 49, 50. The fact that miR-375 targets both ERBB2 and VEGF suggests that it may serve as an upstream target for EC treatment.

Esophageal cancer is composed of two distinct histological subtypes, ESCC and EAC. The two types of EC arise from different cell populations, with ESCC from squamous epithelium and EAC from intestinal metaplastic epithelial cells or Barrett's esophagus. Although miR-375 was considered as a tumor suppressor in the majority of cancers, the role of miR-375 in tumorigenesis is still controversial. For example, miR-375 expression was significantly up-regulated in adenocarcinoma and small cell lung carcinoma but down-regulated in squamous cell carcinoma 26. In our study, the tumor suppressor function of miR-375 was not significantly different between ESCC and EAC, suggesting a broad-spectrum tumor suppressor effect in EC.

In conclusion, this study provides evidence that miR-375 is a tumor suppressor which is frequently suppressed in both EC and precancerous lesions. Decreased miR-375 is associated with overexpression of both ERBB2 and VEGF oncogene genes. These findings suggest that miR-375 may serve as a novel target for EC treatment.

## Figures and Tables

**Figure 5:**
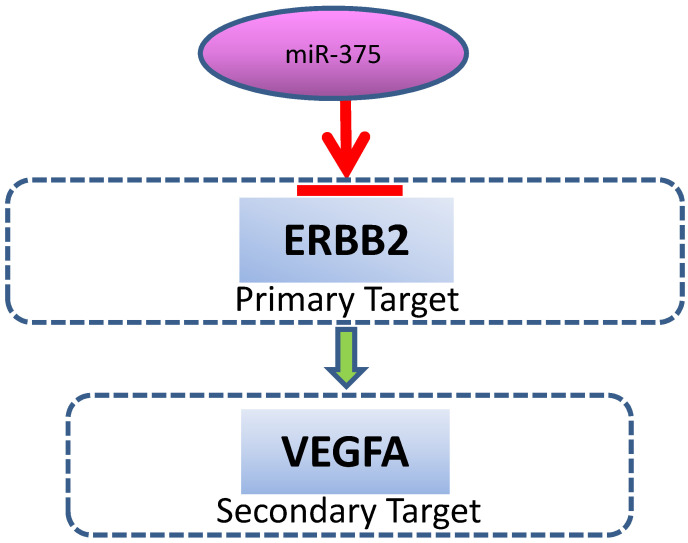
** A schematic model for the regulation of miR-375.** miR-375 directly targets ERBB2, resulting in VEGF downregulation.

**Table 1:** Clinical and pathologic parameters of FFPE samples from patient with EC.

Tissues parameters	Number
**Gender**	
Male	46
Female	11
**Age (Years)**	
31-60	26
61-89	31
**Tumor site**	
Upper third	17
Middle third	18
Lower third	13
Esophagogastric junction	8
Unknown	1
**Metastasis**	
Non-metastasis	18
Metastasis	39
** # of Lymph nodes involved**	
1	3
2-5	11
6-10	25
**Differentiation status**	
Well	13
Moderate	18
Poor	24
Undifferentiated	1
Other	1
**Depth of Invasion**	
Mucosa	4
Submucosa	5
Muscularispropria	18
Serosa/full thickness	30
